# Prognostic significance of lactate dehydrogenase and its impact on the outcomes of gastric cancer: a systematic review and meta-analysis

**DOI:** 10.3389/fonc.2023.1247444

**Published:** 2023-09-01

**Authors:** Jinjin Chen, Xiaoyue Zou

**Affiliations:** Department of Clinical Laboratory, First affiliated Hospital of Huzhou University, Huzhou, China

**Keywords:** lactate dehydrogenase, gastric cancer, prognosis, overall survival, disease free survival

## Abstract

**Background:**

The prognostic significance of lactate dehydrogenase (LDH) and its impact on the outcomes of gastric cancer (GC) is still unclear. We assessed the link between the levels of LDH and the overall survival (OS) and disease-free survival (DFS) in GC patients.

**Methods:**

A comprehensive search (both electronic and manual) was carried out in PubMed *via* MEDLINE, Web of Science (WoS), Experta Medical Database (Embase), and CENTRAL (Cochrane Library) databases for citations that evaluated the strength of association between LDH cut-off levels and OS and/or DFS in GC. Pooled hazard ratios (HRs) with 95% confidence intervals (CIs) were calculated using a random-effects model, and heterogeneity was assessed.

**Results:**

Eighteen studies with 5328 patients were included in our review. The overall pooled HR for OS was 1.48 (95% CI: 1.22-1.80) with high heterogeneity (I2 = 86%). Subgroup analyses showed that the link between LDH and OS was more prominent in Caucasian (HR 1.50 95% CI [0.80, 2.81], p=0.21) than in Asian cohorts (HR, 1.51 95% CI [1.21, 1.87], p=0.002). No significant overall association between LDH and OS (HR = 1.12, 95% CI: 0.76-1.65, p = 0.58) was found. Similar subgroup analyses results were reported for the association between LDH and DFS.

**Conclusion:**

In patients with GC, elevated LDH levels may correlate with worse OS and DFS, but the association is not significant. LDH is a significant predictor of OS but not of DFS. Further studies with larger sample sizes and more standardized criteria for defining elevated LDH levels are needed to confirm our findings.

**Systematic review registration:**

https://www.crd.york.ac.uk/prospero, identifier CRD42023412449.

## Introduction

Gastric cancer (GC) is associated with high morbidity and mortality (1, 2). Despite advancements in medical technology and treatment options, the prognosis for patients with gastric cancer remains challenging. The disease often presents at an advanced stage, limiting curative treatment options and leading to high mortality rates. Therefore, identifying new prognostic biomarkers that can accurately predict disease outcomes and guide treatment decisions, is crucial (3–6).

Lactate dehydrogenase (LDH), an enzyme involved in cellular metabolism, was identified as a possible prognostic biomarker for various malignancies, including gastric cancer. However, the prognostic value of LDH in GC still remains controversial (7). While some studies have suggested a significant association between elevated LDH levels and poor prognosis in gastric cancer patients, other investigations have failed to establish a consistent link. These discrepancies in findings may be attributed to several factors, such as variations in patient populations (ethnicity, age, stage of disease), heterogeneity in study designs (retrospective vs. prospective).

Numerous studies suggest that understanding the link between LDH and cancer can shed light on the metabolic reprogramming that occurs in various cancers, providing insights into the biology of tumor growth and progression. The Warburg effect refers to the distinct metabolic behaviour of cancer cells, wherein they exhibit a preference for glycolysis over mitochondrial oxidative phosphorylation, even under aerobic conditions (8, 9). Recent studies that linked oncogenes and metabolic processes suggested that this transition to aerobic glycolysis may promote cancer cell proliferation (8). LDH is a crucial enzyme that converts pyruvic acid to lactic acid and is over-expressed in all types of cancers. Studies show that LDH stimulates survival, migration, and proliferation of tumour cells, and promotes angiogenesis and metastasis in cases of gastric cancer (10). Elevated levels of LDH were linked to poorer prognosis in a number of cancers, including GC (11, 12). However, there is still no consensus on the association of the expression status and function of LDH-A and the prognosis in gastric cancer patients. To the best of our knowledge, there has been no meta-analysis to synthesize the available evidence. The main goal of this study is to evaluate the prognostic significance of LDH in terms of survival and recurrence of GC.

## Materials and methods

### Search strategy

Comprehensive search was done in PubMed *via* MEDLINE, Web of Science (WoS), Experta Medical Database (Embase), and CENTRAL (Cochrane Library) databases. The search strategy included keywords and MeSH terms related to “gastric cancer”, “LDH”, “prognosis”, and “outcomes”. The search strategy used was as follows: (((Gastric Cancer) OR (Gastric carcinoma)) AND ((“lactate dehydrogenase”[All Fields]) OR (LDH))) AND ((Prognosis) OR (Prognostic) OR Survival [MeSH Term]). The search strategy was adapted to the syntax of each database. A manual examination of the citation sections of the identified papers was then done for supplementary research papers. The bibliographies of the relevant articles were thoroughly searched for any possible missed out studies.

### Study selection

Preferred Reporting Items for Systematic Reviews and Meta-analyses (PRISMA) guidelines (13) were followed throughout our review. Titles and the abstracts of potential studies were independently assessed by two reviewers for their relevance. The reviewers then independently screened full texts of shortlisted studies to determine their eligibility for inclusion. Disputes were resolved by discussion.

#### Inclusion criteria

- Observational Studies (both prospective and retrospective) evaluating the link between total LDH levels and GC prognosis.
- Studies containing HR with 95% CI or providing data from which HR and CI can be derived.
- Studies published in English.
- Studies involving human subjects.

#### Exclusion criteria

- Case reports, Case series, editorials, and blogs
- Studies not reporting the association between LDH and prognosis of gastric cancer.
- Studies with insufficient data to calculate HRs or ORs with 95% CIs.
- Studies not published in English.
- Studies not involving human subjects.
- Studies published in non-peer-reviewed journals.

#### Data extraction

The data that were obtained from eligible reports included: authors, year of publication, design, sample size, baseline characteristics (age, sex, ethnicity), analysis method, cut-off values for total LDH, treatment regimen, outcome measures (OS, DFS, recurrence), HRs with 95% CIs, and adjustments for confounding factors. In cases of dispute, consensus was reached by discussion.

#### Method of quality assessment

Quality was independently rated by the reviewers using the Newcastle-Ottawa Scale (NOS) on three domains: participants selection, comparability of the study groups, and assessment of outcome. Each study got a score of 0-9 according to the quality. Score of 7 or more indicated high quality.

#### Data synthesis and analysis

Review Manager 5.4 software (Cochrane Collaboration, UK) was used to conduct the meta-analysis. We used random-effects models to calculate the HRs and 95% CI for OS and DFS. The random-effects model uses the variation between studies to provide an estimate of the effect size. Interstudy heterogeneity was calculated by I^2^ statistic. An I^2^ value > 70% indicated moderate to substantial heterogeneity. Potential sources of substantial heterogeneity were analysed using subgroup analyses. Subgroup analyses based on sample size, analysis method, cut off values, and patient characteristics like age, ethnicity and treatment regimen among the patients were performed. These analyses helped to explore the sources of heterogeneity and to identify potential effect modifiers that may have influenced the strength of association between LDH and gastric cancer progression.

Publication bias was assessed by funnel plots, visual aids that display the effect size of each study against a measure of study precision. Sensitivity analyses were done to determine the effect of individual studies on the overall result.

## Results

### Study selection process

**Figure 1:**
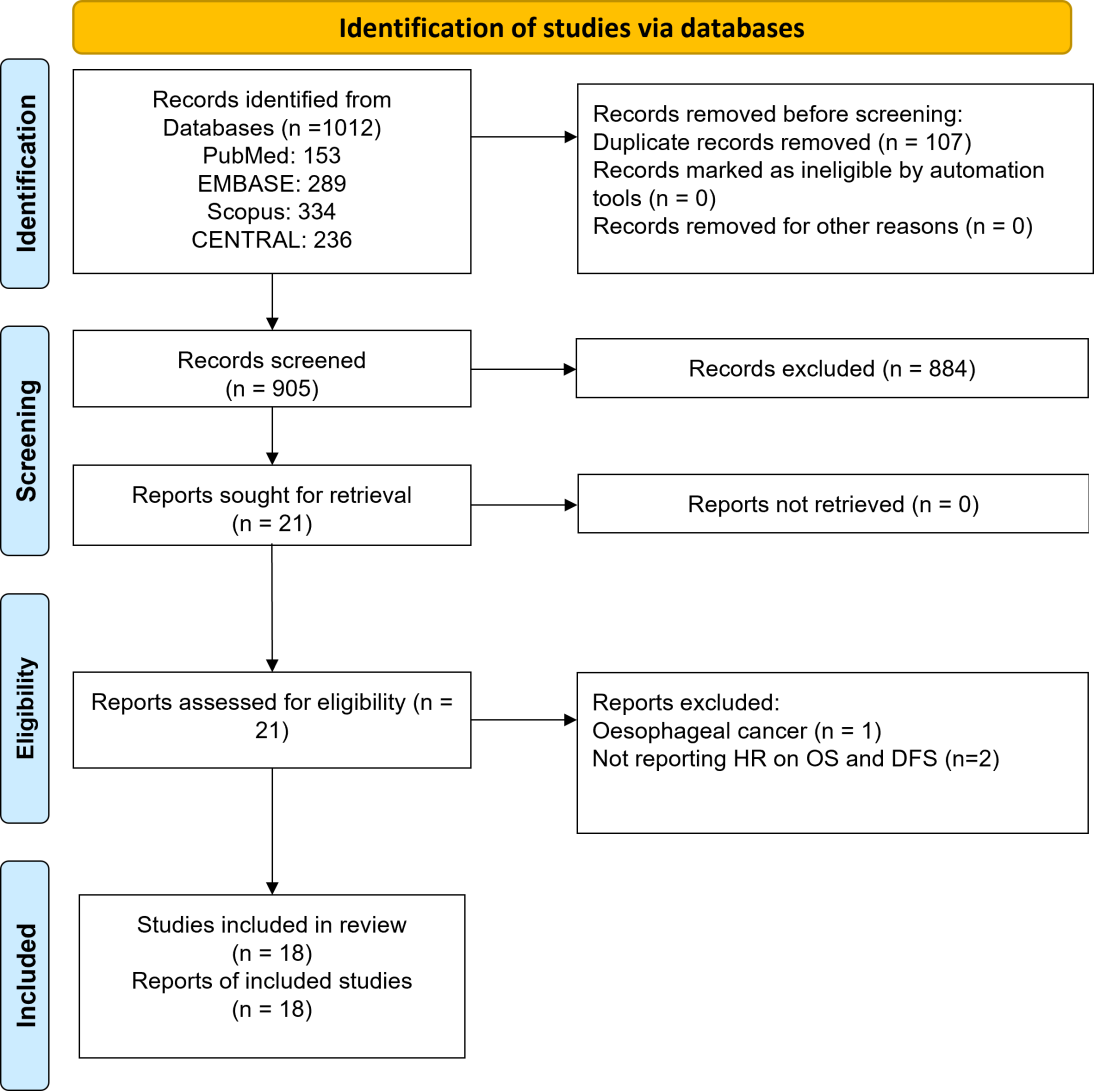
Study Selection Process.

As demonstrated in **Figure 1**, 1012 records were identified using electronic search of all four databases. After removing duplicates, 905 records were subjected to the title and abstract screening. Finally, twenty-one articles were considered potentially eligible and were subjected to full text assessment. Three studies were excluded at that stage: one study reported data on oesophageal cancer and two studies did not report relevant outcomes. Finally, eighteen studies that investigated the prognostic significance of lactate dehydrogenase (LDH) in gastric cancer were included in this review (14–31).

All included studies were retrospective, with the sample sizes ranging from 53 to 868 patients. The studies were done in China (n=8), Japan (n=2), Turkey (n=2), Italy (n=1), Taiwan (n=1), and Greece (n=1). Included papers incorporated univariate analysis (UVA), multivariate analysis (MVA), or both (UVA/MVA). The outcomes assessed were OS, DFS, or both. Patients’ age at diagnosis was 55 to 69 years. LDH cut-off values used varied from the upper limit of normal (ULN) to 480. The treatment strategies used in the studies included resection I, resection + chemotherapy (R+C), immunotherapy (I), and chemotherapy (C), or multiple treatment strategie. (**Table 1**). The overall quality of the studies was average, and a NOS score ranged between 3-7 (**Table 2**).

**Table 1:** Characteristics of the included studies.

Author	Year	Country	Study Design	Ethnicity	Sample Size	Method	Outcome	Age(Years)	LDH Cut-off	Treatment	NOS
Shi et al. (14)	2023	China	Retrospective	Asian	174	UVA/MVA	OS, DFS	63.4	193	Resection	7
Yildirim et al. (16)	2022	Turkey	Retrospective	Caucasian	56	MVA	OS, DFS	61.23 + 8.89	ULN	Resection+ Chemotherapy	7
Chen et al. (15)	2022	China	Retrospective	Asian	146	UVA/MVA	OS, DFS	61	250	Immunotherapy	6
Marshall et al. (19)	2022	Japan	Retrospective	Asian	133	UVA/MVA	OS	65	222	Chemotherapy	7
Hu et al. (18)	2021	China	Retrospective	Asian	61	UVA/MVA	DFS	55.61 + 11.97	299	Immunotherapy	7
Ma et al. (17)	2021	China	Retrospective	Asian	615	UVA/MVA	OS	66	220	Resection	6
Zhou et al. (20)	2020	China	Retrospective	Asian	112	MVA	OS	57	250	Chemotherapy	6
Namikawa et al. (21)	2019	Japan	Retrospective	Asian	262	MVA	OS	69	222	Chemotherapy	4
Fanotto et al. (22)	2017	Italy	Retrospective	Caucasian	868	UVA/MVA	OS, DFS	64	480	Chemotherapy	5
Fuchs et al. (23)	2017	Multiple	Retrospective	Mixed	355	MVA	OS	65	ULN	Chemotherapy	6
Wang et al. (24)	2016	China	Retrospective	Asian	619	MVA	OS, DFS	57.9	ULN	Resection + Chemotherapy	6
Sun et al. (26)	2014	China	Retrospective	Asian	264	UVA/MVA	OS	59	ULN	Resection	6
Turkoz et al. (28)	2014	Turkey	Retrospective	Caucasian	176	UVA	OS	57.8	ULN	Multiple treatment strategies	7
Wang et al. (25)	2014	China	Retrospective	Asian	439	MVA	OS	60	245	None	3
Zhao et al. (27)	2014	China	Retrospective	Asian	365	UVA/MVA	OS, DFS	55	245	Resection	7
Chung et al. (29)	2013	Taiwan	Retrospective	Asian	53	UVA	OS	63.5	ULN	Resection + Chemotherapy	7
Lu et al. (30)	2013	China	Retrospective	Asian	319	MVA	OS	69	ULN	Chemotherapy	4
Sougioultzis et al. (31)	2011	Greece	Retrospective	Caucasian	311	MVA	OS	62	225	Chemotherapy	4

UVA, Univariate Analysis; MVA, Multivariate Analysis; OS, Overall Survival; DFS, Disease Free Survival; LDH, Lactate Dehydrogenase; ULN, Upper limit of Normal.

**Table 2:** Quality Assessment of included studies.

Study	Year	Selection				Comparability	Outcome			Total
		Representativeness of the exposed cohort	Selection of the nonexposed cohort	Ascertainment of exposure	Demonstration that outcome of interest	Basis of the design or analysis	Assessment of outcome	follow-up long enough for outcomes	Adequate follow up	
Shi et al. (14)	2023	1	1	0	1	1	1	1	1	7
Yildirim et al. (16)	2022	1	1	0	1	1	1	1	1	7
Chen et al. (15)	2022	1	0	1	0	1	1	1	1	6
Marshall et al. (19)	2022	1	1	0	1	1	1	1	1	7
Hu et al. (18)	2021	1	1	1	1	0	1	1	1	7
Ma et al. (17)	2021	1	1	0	0	1	1	1	1	6
Zhou et al. (20)	2020	1	1	0	0	1	1	1	1	6
Namikawa et al. (21)	2019	1	0	0	0	0	1	1	1	4
Fanotto et al. (22)	2017	1	1	0	0	0	1	1	1	5
Fuchs et al. (23)	2017	1	1	1	0	0	1	1	1	6
Wang et al. (24)	2016	1	1	1	0	0	1	1	1	6
Sun et al. (26)	2014	1	1	1	0	0	1	1	1	6
Turkoz et al. (28)	2014	1	1	0	1	1	1	1	1	7
Wang et al. (25)	2014	1	1	0	0	0	0	0	1	3
Zhao et al. (27)	2014	1	1	0	1	1	1	1	1	7
Chung et al. (29)	2013	1	1	1	1	0	1	1	1	7
Lu et al. (30)	2013	1	0	0	0	0	1	1	1	4
Sougioultzis et al. (31)	2011	1	0	0	0	0	1	1	1	4

### Meta-analysis

The Hazard ratios (HR), showing the association of elevated LDH enzyme with the OS and DFS of gastric cancer patients, were combined using general inverse variance (GIV) to estimate the overall effect sizes from different studies using RevMan 5.4 v software (Cochrane Collaboration, UK).

### Overall-survival

**Figure 2:**
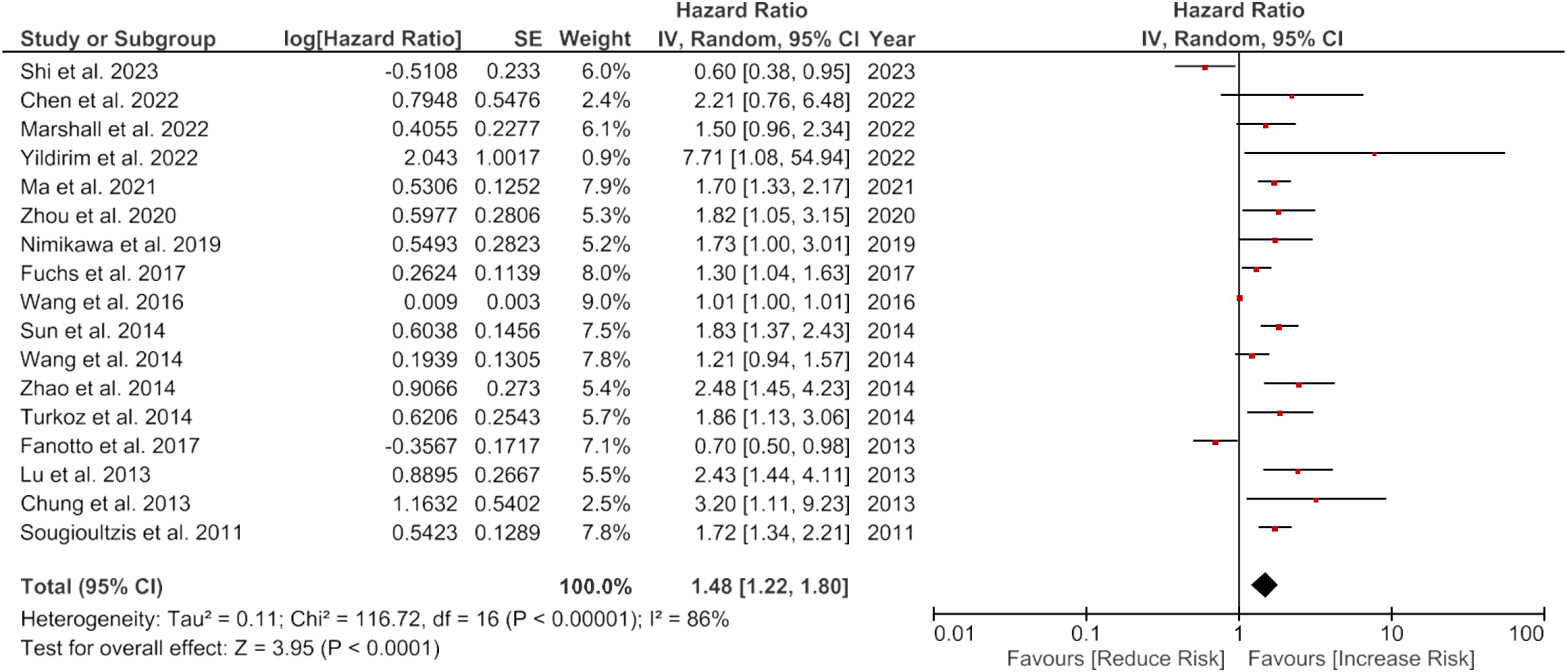
Forest plot showing the association of LDH and overall survival of gastric cancer patients.

The overall meta-analysis showed a pooled HR of 1.48 (95% CI: 1.22-1.80) with high heterogeneity (I2 = 86%, p<0.0001), indicating varying effect sizes of the studies (**Figure 2**).

#### Subgroup analysis

When sub-grouped by ethnicity, the pooled HR was 1.50 (95% CI: 0.80-2.81) for Caucasian, and 1.51 (95% CI: 1.21-1.87) for Asian patients, with high heterogeneity observed in both subgroups (I2 = 87% and I2 = 86%, respectively). Stratification by sample size revealed a higher pooled HR for studies with sample sizes greater than 200 (pooled HR 1.44, 95% CI: 1.16-1.78) comparing to studies with sample sizes less than 200 (pooled HR 1.74, 95% CI: 0.99-3.07). However, both subgroups showed high heterogeneity (I2 = 88% and I2 = 79%, respectively). When stratified by method, the pooled HR was 1.43 (95% CI: 1.17-1.75) for studies using multivariate analysis (MVA) and 1.38 (95% CI: 0.86-2.23) for studies using univariate analysis (UVA), with high heterogeneity observed in both subgroups (I2 = 87% and I2 = 93%, respectively).

Stratification by treatment showed a higher pooled HR for studies using chemothIpy (C) (pooled HR 1.46, 95% CI: 1.10-1.95) compared to studies using rIction (R) (pooled HR 1.30, 95% CI: 0.91-1.87) or resection with chemotherapy (R+C) (pooled HR 2.23, 95% CI: 0.70-7.13). A lower pooled HR was observed for studies using immunotherapy (I) (pooled HR 1.92, 95% CI: 1.22-3.01), but this subsection included only two studies. There was high heterogeneity in the C subgroup (I2 = 76%) and R+C subgroup (I2 = 77%), but not in the R (I2 = 85%) or I (I2 = 0%) subgroups.

Finally, stratification by age showed a higher pooled HR for studies with patients > 60 years old (pooled HR 1.46, 95% CI: 1.11-1.92) compared to studies with patients < 60 years old (pooled HR 1.55, 95% CI: 1.13-2.12), with high heterogeneity observed in both subgroups (I2 = 78% and I2 = 87%, respectively).

The subgroup analysis based on GC subtypes demonstrated a significant association between increased LDH levels and worse overall survival in Gastric Adenocarcinoma patients, with HR of 1.72 (95% CI: 1.30-2.29), indicating that patients with elevated LDH levels had a 72% higher risk of experiencing worse overall survival compared to patients with normal LDH levels (**Table 3**).

**Table 3:** Sub-group analysis of association of LDH with overall survival in gastric patients.

		Studies	HR (95% CI)	Weight	I^2^	X^2^	*p* value
Overall		17	1.48 [1.22-1.80]	100%	86%	116.72	<0.0001
Ethnicity							
	Caucasian	4	1.50 [0.80, 2.81]	21.50%	87%	22.77	0.21
	Asian	13	1.51 [1.21, 1.87]	78.50%	86%	85.25	0.0002
Sample Size							
	¾200	7	1.74 [0.99, 3.07]	28.90%	79%	24.09	0.05
	>200	10	1.44 [1.16, 1.78]	71.10%	88%	83.5	0.001
Method							
	UVA	8	1.38 [0.86, 2.23]	17.70%	93%	96.41	0.18
	MVA	9	1.43 [1.17, 1.75]	82.50%	87%	117.8	0.0005
Treatment							
	R	4	1.30 [0.91, 1.87]	26.80%	85%	19.36	0.16
	C	7	1.46 [1.10, 1.95]	45.10%	76%	25.08	0.009
	R+C	3	2.23 [0.70, 7.13]	11.20%	77%	8.69	0.18
	I	2	1.92 [1.22, 3.01]	8.10%	0%	0.08	0.005
Age							
	¾60	6	1.55 [1.13, 2.12]	40.70%	87%	39.65	0.006
	>60	11	1.46 [1.11, 1.92]	59.30%	78%	45.79	0.008
Tumor Type							
	Adeno-carcinoma	10	1.72 [1.30,2.29]		88%	74.99	0.0002
	Lymphoma	1	3.20 [1.11,9.23]		NA	NA	0.03
	Mixed	6	1.13 [0.79,1.61]		84%	31.01	0.50

### Disease free survival

**Figure 3:**
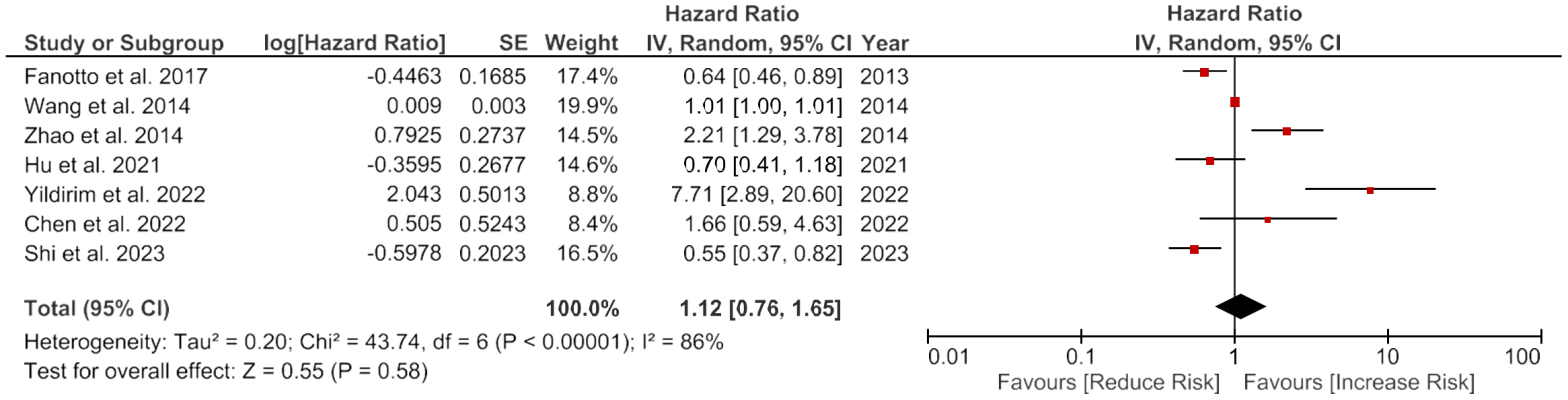
Forest plot showing the association of LDH and disease-free survival of gastric cancer patients.

There was no significant overall link between the higher LDH enzyme levels and the DFS (HR = 1.12, 95% CI: 0.76-1.65, p = 0.58). However, there was a considerable heterogeneity among the studies, with an I2 value of 86% (**Figure 3**).

#### Subgroup analysis

There was a non-significant positive association between LDH and DFS in Caucasian patients (HR = 2.12, 95% CI: 0.19-24.32, p = 0.54) and no association observed in Asian patients (HR = 1.00, 95% CI: 0.67-1.49, p = 1.00). Studies with smaller sample sizes had a positive but not meaningful association (HR = 1.36, 95% CI: 0.52-3.56, p = 0.53), whereas studies with larger sample sizes had no association (HR = 1.06, 95% CI: 0.67-1.69, p = 0.79). Univariate analysis (UVA) and multivariate analysis (MVA) showed similar results. The subgroup analysis based on the treatment showed a non-significant positive association in patients receiving intervention C (HR = 1.46, 95% CI: 1.10-1.95, p = 0.009), whereas patients receiving intervention R or R+C had no significant association. The subgroup analysis based on age showed no marked difference between patients aged <60 years and patients over 60 years old (**Table 4**).

**Table 4:** Sub-group analysis of association of LDH with disease free survival in gastric patients.

		Studies	HR (95% CI)	Weight	I^2^	X^2^	*p* value
Overall		7	1.12 [0.76, 1.65]	100%	86%	43.74	0.58
Ethnicity							
	Caucasian	2	2.12 [0.19, 24.32]	26.20%	95%	22.15	0.54
	Asian	5	1.00 [0.67, 1.49]	73.80%	80%	19.98	1
Sample Size							
	¾200	4	1.36 [0.52, 3.56]	48.20%	88%	26.05	0.53
	>200	3	1.06 [0.67, 1.69]	51.80%	87%	15.5	0.79
Method							
	UVA	4	1.03 [0.54, 1.97]	41.50%	89%	26.22	0.94
	MVA	7	1.12 [0.76, 1.65]	58.50%	86%	43.74	0.58
Treatment							
	R	2	1.09 [0.28, 4.25]	31%	94%	16.69	0.9
	I	2	0.96 [0.42, 2.16]	23%	54%	2.16	0.91
Age							
	¾60	3	1.13 [0.69, 1.86]	49%	80%	10.09	0.62
	>60	4	1.28 [0.55, 2.97]	51%	89%	27.12	0.57
Tumor Type							
	Adeno-carcinoma	2	1.01 [1.00, 1.02]		0%	0.89	0.003
	Mixed	5	1.20 [0.60, 2.39]		90%	39.50	0.60

**Figure 4:**
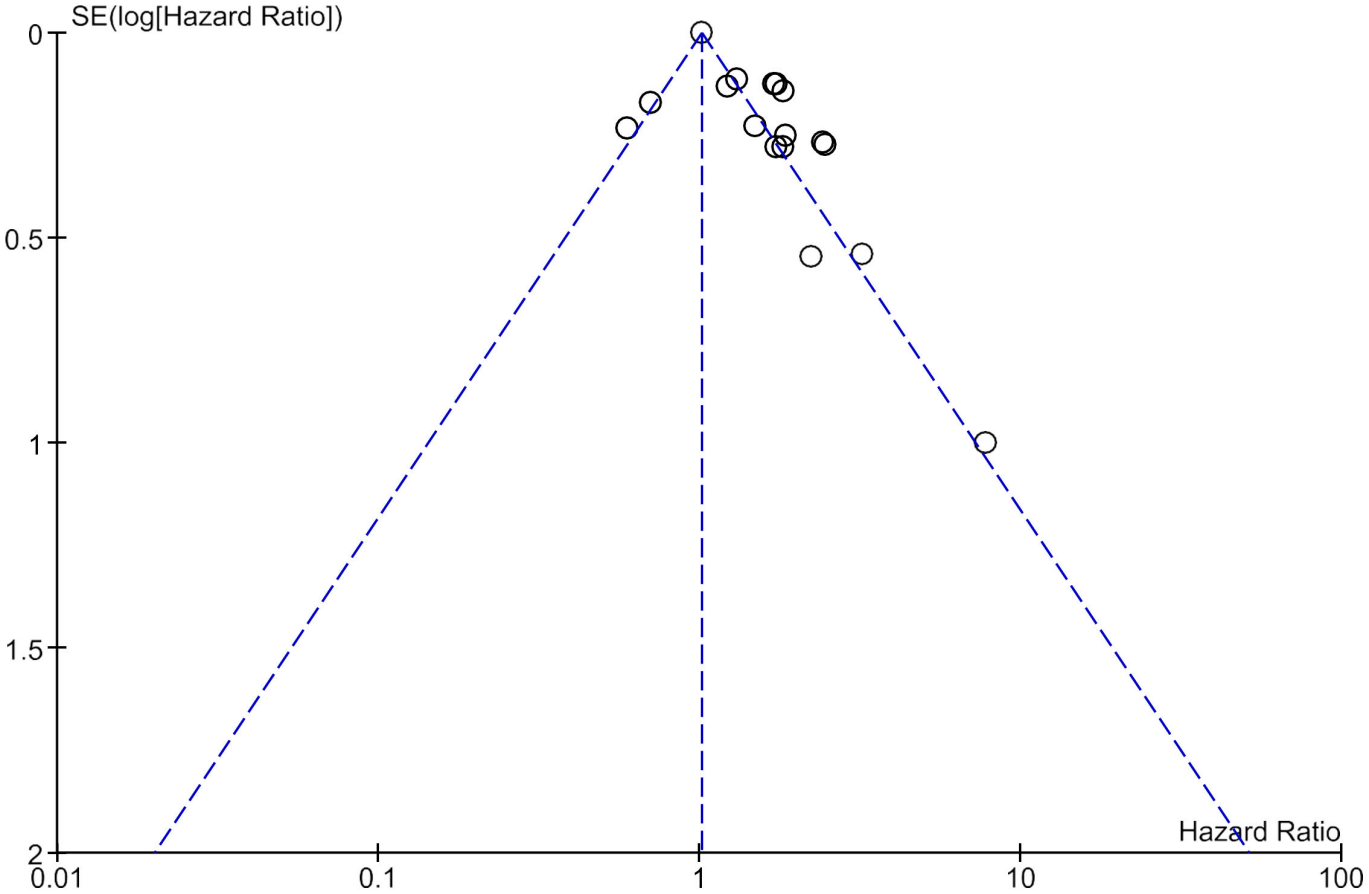
Funnel plot of all included studies showing no publication bias in studies assessing Overall survival.

A sensitivity analysis was carried out by carefully removing each included studies from the forest plots showing the association of LDH with OS and DFS. No significant change in the effect estimate was noticed in the combined HR of OS and DFS. This indicates that there is no effect of the weight of any included studies. No publication bias was detected on the visual examination of funnel plots constructed for overall survival (**Figure 4**).

## Discussion

GC is one of the most prevalent forms of cancer worldwide, and remains a significant health concern due to its poor prognosis and high mortality rates (2). Lactate dehydrogenase (LDH) is crucial for energy metabolism (10), and its levels have been shown to be associated with all types of cancers (7). Our results showed positive association of elevated LDH levels with poor OS in gastric cancer patients. The overall HR for OS was 1.48 (95% CI: 1.22-1.80), indicating that in GC patients, elevated LDH levels are associated with a 48% greater mortality risks. Additionally, the HR for DFS did not show any association. There was a substantial variability among studies (I2 value of 86%). However, the subgroup analysis based on ethnicity, sample size, method, treatment, and age did not reveal significant differences in the results, suggesting that LDH levels are a robust prognostic factor for GC patients in terms of overall survival of the patients.

The link between elevated LDH levels and poor prognosis in gastric cancer patients can be explained by the fact that LDH is involved in anaerobic metabolism, which is commonly observed in cancer cells due to their increased glycolytic activity (10, 32, 33). This process produces high levels of lactate, which is converted back to pyruvate by LDH. The elevated LDH levels reflect the increased glycolytic activity and indicate the presence of more aggressive cancer cells that are resistant to chemotherapy and radiotherapy, leading to poor clinical outcomes (7).

Our findings have important clinical implications. Firstly, measuring LDH levels may help identify patients with a potentially poor prognosis and enable clinicians to develop more personalized treatment plans. Patients with high LDH levels may require more aggressive treatments, such as chemotherapy or targeted therapy, to improve their survival outcomes (11, 22). Secondly, LDH can be regarded as a potential biomarker to monitor response to intervention and disease progression (34). A decrease in LDH levels after treatment may indicate a favourable response and a reduced risk of disease recurrence (35).

LDH exists as five isoenzymes, each with distinct tissue distributions and functions. While our analysis included studies reporting total LDH levels, considering specific isoenzymes could offer valuable insights into the differential expression patterns and their relevance to GC outcomes. Although our meta-analysis did not directly examine individual LDH isoenzymes due to limited data availability in the included studies, future investigations focusing on these specific isoenzymes may shed light on their unique roles in gastric cancer pathogenesis and prognosis. Such studies could help unravel the underlying mechanisms linking LDH activity to tumor biology and may potentially lead to the identification of more precise prognostic markers for distinct subtypes of GCs.

The subgroup analysis based on GC types revealed varying associations between elevated LDH levels and survival outcomes. In gastric adenocarcinomas, elevated LDH levels were consistently linked to worse survival outcomes. The metabolic alterations associated with increased LDH activity may play a crucial role in the aggressive behaviour of gastric adenocarcinomas, contributing to tumour growth and progression. However, the availability of data limited our ability to perform subgroup analysis for some GC subtypes, emphasizing the need for more extensive and well-designed studies in these areas.

This review has many strengths, such as including studies with large sample size and the inclusion of multiple studies, which increase the generalizability and reliability of the results. However, there are also some limitations to consider. Firstly, the high heterogeneity that we observed may affect the robustness of the findings. Secondly, the quality of the incorporated studies varied, and some had a significant bias risk. Lastly, the lack of data on the HR for DFS limits the interpretation of the results and their clinical implications.

To conclude, we reported a significant association of high LDH levels with poor OS in GC patients. LDH was a significant predictor of OS but not of DFS. LDH, therefore, can be used as a valuable prognostic factor on GC patients, and may facilitate the development personalized treatment plans. Future studies are needed to address the limitations of our meta-analysis, such as reducing heterogeneity and including more high-quality studies with data on DFS, to provide more robust evidence on the prognostic significance of LDH in gastric cancer.

## Data availability statement

Publicly available datasets were analyzed in this study. This data can be found here: Comprehensive search was done in online medical databases PubMed, Web of Science (WoS), Experta Medical Database (Embase), and CENTRAL (Cochrane Library).

## Author contributions

JC and XZ conceived and designed the study. JC and XZ collected the data and performed the analysis. XZ was involved in the writing of the manuscript and is responsible for the integrity of the study. All authors contributed to the article and approved the submitted version.
